# The Role and Involvement of Functional Three-Dimensional Porcine-Derived Collagen Matrix Biomaterials in Periodontal Regeneration: A Comprehensive Review

**DOI:** 10.3390/jfb16110417

**Published:** 2025-11-07

**Authors:** Cristian Cojocaru, Dana Gabriela Budala, Dragos Ioan Virvescu, Gabriel Rotundu, Florinel Cosmin Bida, Teona Tudorici, Zinovia Surlari, Mihaela Scurtu, Ancuta Goriuc, Ionut Luchian

**Affiliations:** Grigore T. Popa University of Medicine and Pharmacy, 700115 Iasi, Romania

**Keywords:** porcine-derived biomaterials, three-dimensional scaffolds, collagen matrix, functional soft-tissue regeneration, periodontal surgery

## Abstract

Three-dimensional porcine matrix-derived biomaterials have emerged as valuable tools in periodontal regeneration, offering structural stability, biocompatibility, and favorable cellular responses. This review summarizes their physicochemical characteristics, biological mechanisms, and clinical performance in guided tissue and bone regeneration. Comparative analyses show superior handling, integration potential, and regenerative predictability compared with collagen and synthetic scaffolds, especially in complex intrabony and furcation defects. Despite promising clinical outcomes, heterogeneity in processing techniques and limited long-term data still hinder standardization. Overall, porcine-derived scaffolds represent reliable and biologically active options for periodontal regeneration. Future innovation focusing on functionalization, cell integration, and patient-tailored design will define the next generation of predictable and biomimetic regenerative solutions.

## 1. Introduction

Periodontal disease remains one of the most prevalent chronic conditions worldwide, leading to progressive destruction of the supporting structures of the teeth and ultimately to tooth loss if untreated [1]. While guided tissue regeneration (GTR), scaling and root planning, and other conventional therapy can slow the advancement of illness, they seldom succeed in regenerating periodontal tissues in a way that is both thorough and predictable [2]. This gap in treatment has prompted researchers to look for biomaterials that can restore the alveolar bone, periodontal ligament, and cementum that make up the periodontal architecture in a way that is biologically functional [3]. Conventional approaches, such as guided tissue regeneration (GTR), bone grafting, and the use of barrier membranes, have provided partial success but are often limited by unpredictable outcomes [4,5]. Consequently, there is a growing demand for regenerative strategies that are both biologically reliable and clinically reproducible [4].

Matrixes generated from porcine have attracted a lot of interest among the biomaterials created for periodontal regeneration. These materials provide a natural three-dimensional scaffold that facilitates cell adhesion, migration, and differentiation [6,7,8]. They replicate the structure and composition of the extracellular matrix (ECM). They are a great substitute for synthetic or allograft scaffolds because of their mechanical qualities, good resorption profile, and biocompatibility [9,10].

Clinically, 3D porcine matrices are used across a spectrum of periodontal and peri-implant indications. In intrabony defects, they may serve as barrier-scaffolds (alone or over particulate grafts) to maintain space and stabilize the wound; in class II furcation, they can aid in compartmentalization and clot protection where root divergence challenges membrane adaptation; in mucogingival surgery, volumetric collagen matrices offer an alternative to autogenous connective tissue grafts for recession coverage and soft-tissue thickening; and in the peri-implant context, they support mucosal phenotype modification, ridge preservation, and guided bone regeneration [11,12].

Their handling characteristics—hydration behavior, suture-holding capacity, compressibility, and adaptability to complex morphologies—are central determinants of intraoperative workflow and outcomes.

While the application of porcine ECM-derived biomaterials in periodontal and peri-implant regeneration has significantly increased, the existing research is nevertheless diverse and frequently constrained by variations in material processing, defect characteristics, and clinical protocols [13]. The molecular mechanisms by which these scaffolds affect healing—specifically immune response regulation, angiogenesis, and long-term tissue integration—are not fully elucidated, and comparative data with other biomaterials is limited.

The present comprehensive narrative review aims to summarize and interpret current biological, preclinical and clinical knowledge regarding the role and involvement of three -dimensional porcine matrix-derived functional biomaterials in periodontal regeneration. This study aims to offer a current summary of the potential, constraints, and future trajectories of regenerative periodontal therapy by synthesizing data from experimental and clinical investigations.

## 2. Literature Review

Despite the growing interest in regenerative materials, most previous reviews have primarily focused on synthetic polymers, allogenic grafts, or generic collagen scaffolds. However, the specific contribution of three-dimensional porcine ECM scaffolds to periodontal regeneration remains underexplored, particularly regarding their biological mechanisms, structural optimization, and long-term clinical outcomes. Therefore, a comprehensive synthesis of current evidence is necessary to clarify their regenerative potential and identify existing gaps in translational applicability.

This paper was conceived as a comprehensive narrative review, aiming to synthesize current knowledge and clinical evidence concerning the role of three-dimensional porcine matrix–derived biomaterials in periodontal regeneration. By integrating findings from basic science, animal studies, and clinical applications, this work seeks to provide an updated perspective on their biological potential, therapeutic efficacy, and future directions in regenerative periodontology.

**Figure 1:**
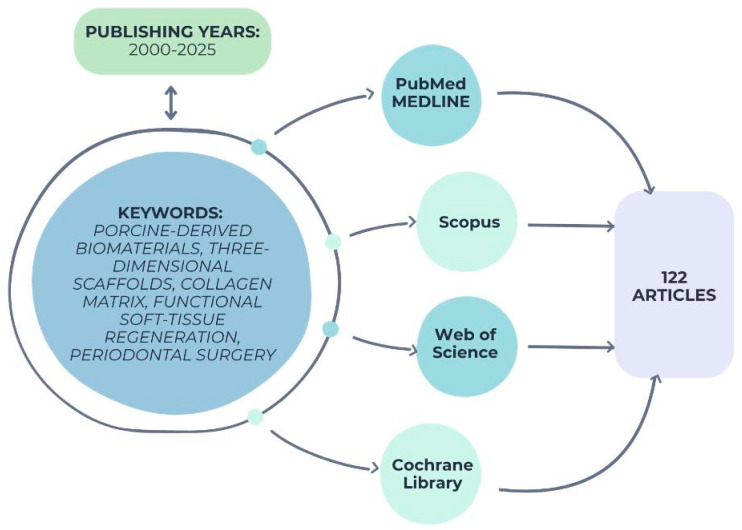
Search strategy and selection workflow for the reviewed literature.

The evidence discussed herein originates from peer-reviewed publications retrieved from major biomedical databases, including PubMed/MEDLINE, Scopus, Web of Science, and the Cochrane Library. Relevant studies published between 2000 and 2025 were considered to capture both foundational and emerging concepts in porcine extracellular matrix (ECM) applications. The following keywords and Boolean operators were used: (“porcine” OR “xenogeneic”) AND (“collagen matrix” OR “extracellular matrix” OR “ECM” OR “SIS” OR “acellular dermal matrix”) AND (“periodontal regeneration” OR “guided tissue regeneration” OR “soft tissue augmentation” OR “gingival recession” OR “peri-implant”. Manual cross-referencing of cited literature within key papers was performed to ensure inclusion of landmark studies and significant clinical trials, as in Figure 1 below:

Inclusion Criteria

Studies were included if they met the following conditions:

✓ Investigated porcine-derived 3D extracellular matrix biomaterials (e.g., collagen matrix, small intestinal submucosa, pericardial, or dermal scaffolds);
✓ Evaluated their application in periodontal or peri-implant tissue regeneration (hard or soft tissue);
✓ Reported quantitative or qualitative outcomes such as clinical attachment level (CAL) gain, probing depth (PD) reduction, bone fill, root coverage, or soft tissue thickness.
✓ Included preclinical (in vitro or animal) or clinical (randomized, cohort, or case series ≥ 5 patients) designs.
✓ Published in English in peer-reviewed journals.

The selection process focused on studies involving porcine-derived collagen or ECM scaffolds used for periodontal or peri-implant soft- and hard-tissue regeneration. Preclinical models, in vitro mechanistic investigations, and human clinical trials (randomized and observational) were included to form a multidimensional understanding of biomaterial function and performance. Publications were considered relevant when they provided histologic, radiographic, or clinical outcomes relating to defect fill, attachment gain, or soft-tissue volume enhancement.

Exclusion Criteria

✓ Studies using non-porcine or synthetic scaffolds without ECM origin.
✓ Case reports with <5 patients, letters, editorials, or conference abstracts.
✓ Publications without measurable outcomes or with insufficient methodological clarity.

Data from all eligible publications were systematically charted to capture study design, material characteristics, intervention protocol, clinical indication, follow-up duration, and primary outcomes related to periodontal or peri-implant regeneration. Given the heterogeneity of the available data, the narrative design of this review does not allow for quantitative pooling or meta-analysis. Differences in material composition, study design, and clinical application limit direct comparison between studies. A qualitative synthesis was performed by thematic grouping of findings into: (1) biological and bioengineering foundations; (2) preclinical evidence; (3) clinical outcomes according to defect type; (4) safety and complications; and (5) comparative performance versus autogenous grafts and other xenogeneic matrices.

Although this review follows a narrative framework, a structured appraisal process was used to ensure methodological transparency. Articles were screened independently by the authors for relevance, quality, and completeness of data. Study quality was evaluated according to study type: randomized clinical trials were examined for randomization, sample size calculation, blinding, and outcome reporting consistency; preclinical animal studies were assessed for ethical approval, control group design, and histologic reproducibility; and in vitro studies were evaluated for material characterization, replicability, and standardization of assays.

To weigh the strength of evidence, priority was given to systematic reviews, meta-analyses, and randomized clinical trials, followed by well-designed controlled preclinical studies and mechanistic in vitro research. Discrepancies between studies were qualitatively analyzed considering differences in defect model, follow-up duration, and scaffold composition. The narrative synthesis emphasized convergence of findings across study types and their clinical translation potential, thereby providing an evidence-informed overview despite heterogeneity in study designs.

After identifying and analyzing the relevant body of evidence, it becomes essential to understand the biological rationale and structural design that make three-dimensional porcine ECM scaffolds suitable for periodontal and peri-implant regeneration.

### 2.1. Biological and Bioengineering Fundamentals of Porcine ECM Scaffolds

Understanding the biological and engineering fundamentals of porcine ECM scaffolds is essential to elucidate how these materials support periodontal regeneration.

Their regenerative potential derives from a combination of native extracellular matrix composition, preserved ultrastructure, and bio functional cues that interact with host cells and signaling pathways to promote constructive tissue remodeling.

An essential non-cellular macromolecular three-dimensional network of macromolecules, the ECM plays a key function in regulating homeostasis and tissue development [14,15]. The primary components of ECM are water, (glyco)proteins, proteoglycans (PGs), and heteropolysaccharides like hyaluronan. Nevertheless, in order to preserve its own and the organ’s structural integrity, each kind of tissue develops its own specific matrix composition and architecture [16,17].

Despite its reputation as an ostensible observer of biological events, extracellular matrix quietly guides cellular actions from the beginning of embryogenesis all the way into adulthood, regulating the growth of tissues and maintaining internal stability [18]. Matrix macromolecules do more than just hold cells in place; they also produce biochemical and biomechanical signals that regulate cell shape, signaling, spatial organization, and communication between cells [19].

Collagen, elastin, laminin, fibronectin, proteoglycans (PGs), and glycosaminoglycans (GAGs) are the primary components of the extracellular matrix (ECM) [20]. To further tissue engineering applications, it is crucial to synthesize ECM-based platforms, since ECM macromolecules play a preponderant role in tissue formation and a number of pathological states [21]. Given the predominant role of ECM macromolecules in both tissue development and various pathological conditions, the synthesis of ECM-based platforms is of critical importance for advancing tissue engineering applications.

There are three primary types of ECM-based scaffolds used in tissue engineering: natural, synthetic, and hybrid, which are dependent on the origin of the monomers used [22].

Because they are bio-based and chemically similar to native extracellular matrix components, natural scaffolds maintain the structural integrity and biochemical signals necessary for mediating cellular processes [23]. On the flip side, lab-engineered polymer synthetic scaffolds allow for fine-grained regulation of mechanical qualities like porosity, stiffness, elasticity, and strength. Hybrid composites, which combine synthetic and natural ECM components, combine the bioactivity and mechanical strength of biological components, making them a potential solution for a range of tissue engineering and regenerative medicine applications [24].

Porcine-derived extracellular matrix scaffolds are predominantly composed of type I and type III collagen, with minor fractions of elastin and glycosaminoglycans that preserve the mechanical and biochemical attributes of native connective tissue [25]. Depending on the source tissue—dermis, small intestinal submucosa (SIS), or pericardium—the matrices exhibit distinct fibrillar orientations, density profiles, and mechanical behavior [25].

Dermal matrices present compact collagen bundles suitable for volumetric soft-tissue reconstruction; SIS scaffolds display a highly porous and interconnected network that favors vascular infiltration and cellular repopulation; pericardial matrices offer thin, tensile lamellae providing dimensional stability under tension [26].

This native-like composition and architecture confer intrinsic biomimetic properties, enabling the scaffold to sustain cell attachment and migration while guiding early tissue remodeling [27]. Preservation of the collagen triple-helix structure and native cross-linking patterns ensures adequate tensile strength and a controlled degradation profile, both critical for predictable regenerative outcomes [27].

The regenerative behavior of porcine ECM scaffolds is largely determined by the efficiency of the decellularization and sterilization processes, which aim to eliminate immunogenic cellular remnants while preserving the structural and biochemical integrity of the matrix [28].

A major obstacle in tissue engineering is the development of bio scaffolds that minimize host immunological rejection while closely imitating the structure of original tissues. This is because unfavorable immune responses can occur [29]. The use of dECM scaffolds in tissue engineering and regeneration is gaining traction as a potential strategy for eradicating immune-related problems [30,31]. Natural bio scaffolds for engineering and regeneration techniques can be created through decellularization, which involves eliminating cells and their components while maintaining the native ECM structural and functional microenvironmental properties [32].

Importantly, in order to decrease inflammatory and immune responses, it is necessary to remove antigens and cellular components from tissues. On the other hand, growth factors and structural macromolecules of the matrix mediate the functional properties of cells, such as adhesion, proliferation, differentiation, and migration [33,34,35].

Persistent damage-associated molecular patterns (DAMPs) due to incomplete decellularization might cause inflammation to continue and activate macrophages that are pro-inflammatory (M1-like) during inflammation [36]. In contrast, the regenerative capacity of the scaffold can be diminished if it is over-processed, as this can lead to the degradation of important ECM proteins, growth factors, and biomechanical characteristics [37].

**Figure 2:**
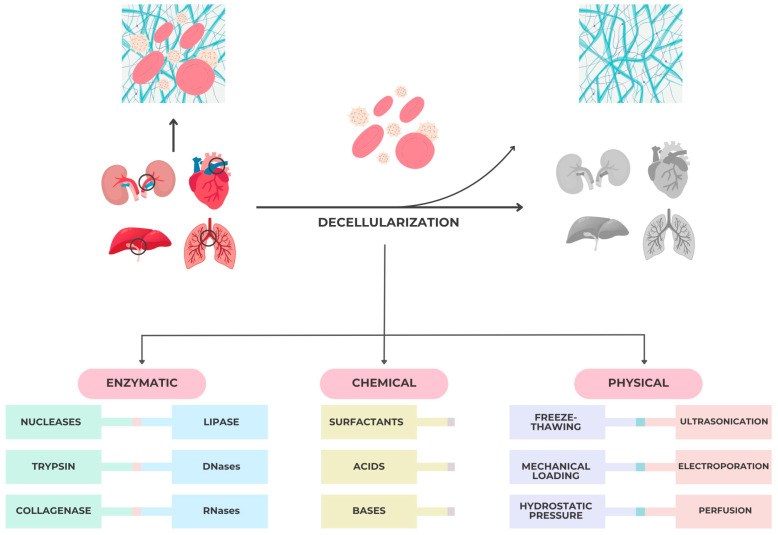
Major tissue/organ decellularization techniques for bio scaffolds fabrication.

Chemical, enzymatic, and physical techniques are employed in various combinations to achieve this balance. Typical protocols involve mild detergents or enzymatic digestion to remove cellular components, followed by repeated rinsing cycles to minimize residual agents that may alter collagen stability. As illustrated in Figure 2, the decellularization techniques used to fabricate porcine ECM scaffolds vary in their mechanism and impact on tissue integrity. Chemical agents such as SDS ensure efficient cellular removal but can disrupt collagen stability, while enzymatic and physical methods preserve the bioactivity of ECM. This balance between effective decellularization and matrix preservation is fundamental for ensuring host biocompatibility and constructive remodeling in periodontal applications.

Decellularization protocols typically integrate chemical, enzymatic, and physical approaches to ensure the complete removal of cellular remnants while maintaining the biochemical and structural integrity of the extracellular matrix (ECM).

Among chemical agents, surfactants are most frequently employed and are classified according to their charge as ionic, non-ionic, or zwitterionic [38]. Ionic surfactants such as sodium dodecyl sulfate (SDS), sodium deoxycholate, or Triton X-200 effectively disrupt cell membranes, solubilize lipids, and release cytoplasmic material; however, excessive exposure can impair collagen fibrillar integrity, decrease glycosaminoglycan (GAG) content, and alter growth-factor binding capacity [38,39,40,41,42]. Non-ionic surfactants, including Triton X-100, act more gently on the ECM structure but can still interfere with DNA–protein interactions and compromise mechanical stability if used at high concentrations [29,32].

In addition to surfactants, acidic and alkaline reagents facilitate cellular lysis and nucleic acid degradation. Because extreme pH conditions may induce denaturation or disorganization of collagen fibers, the concentration and exposure time must be carefully optimized [39,40].

Enzymatic decellularization represents a complementary strategy that enhances cellular removal while preserving bioactivity. Enzymes such as nucleases, collagenases, trypsin, lipase, and dispase degrade residual DNA and cytoplasmic fragments, supporting a cleaner scaffold microenvironment. Nevertheless, over-digestion—particularly with proteolytic agents like trypsin—can reduce key ECM components and weaken the mechanical framework [32,43].

Therefore, achieving the desired balance between complete cell removal and matrix preservation requires a precise combination of agents, sequential washing steps, and standardized quality controls. When optimized, these methods produce bioactive porcine ECM scaffolds capable of maintaining native ultrastructure, biochemical signaling domains, and mechanical stability necessary for periodontal tissue regeneration.

Physical decellularization techniques are typically utilized with chemical and enzymatic methods. Some of the most common methods are mechanical stress, freeze–thaw, hydrostatic pressure, ultrasonication, electroporation, and perfusion.

Decellularized extracellular matrix (ECM) scaffolds depend on a fragile balance to draw in endogenous stem/progenitor cells, send out bioactive signals, and encourage a type 2 immune response that is pro-remodeling. It is important to save ECM components that change the immune system, but cell remnants that cause immunological responses should be thrown away. Once cell removal has been verified, it is essential to assess the impact of decellularization on the mechanical properties of the remaining ECM scaffold. The type of mechanical testing that should be utilized should depend on what the clinical application needs [44]. Standardized techniques customized to particular tissues are necessary for reproducibility and effective clinical translation.

The diversity of decellularization methods reflects the ongoing challenge of reconciling efficient cellular removal with the preservation of extracellular matrix functionality. No single protocol achieves an ideal outcome across all tissue types; therefore, the success of porcine ECM preparation depends on method optimization tailored to tissue origin and intended clinical use, ensuring that bioactivity and mechanical integrity are maintained throughout processing [45].

ECM scaffolds have demonstrated their usefulness as an inductive template for the development of novel tissues in both pre-clinical animal models and human therapeutic applications.

Preservation of the native collagen architecture and bioactive molecules, such as growth factor–binding domains and adhesion peptides, is essential to maintain biological functionality. Excessive processing can denature collagen fibrils and reduce the matrix’s capacity to support cellular repopulation and angiogenesis, whereas insufficient decellularization may trigger inflammatory responses [46].

The goal of sterilization is to eliminate the possibility of contamination by living microorganisms, such as viruses, yeasts, and bacteria. For biodegradable scaffolds to continue serving their intended function after sterilization, the sterilization method must preserve the scaffolds’ structural and biochemical characteristics. Still, there are several downsides to these procedures and Table 1 provides an overview of the microbial inactivation capacity of major sterilization techniques. Heat and gamma irradiation achieve complete sterilization but compromises the mechanical and biochemical integrity of collagen scaffolds, while plasma and EtO methods balance efficacy with biocompatibility, making them more suitable for clinical-grade biomaterials.

For decades, researchers have tried to sterilize biodegradable scaffolds using the same methods used in clinical settings, including ethylene oxide (EtO) and gamma irradiation, but thus far, the results have been mixed. As detailed in Table 2, each sterilization method has distinct advantages and drawbacks. For porcine ECM scaffolds, chemical and irradiation-based approaches require careful optimization to avoid denaturation of native collagen, which would impair vascularization and tissue integration.

Because of their unique chemical characteristics, biodegradable scaffolds are especially vulnerable to the kinds of conditions necessitated by conventional sterilization procedures. For this reason, optimization of sterilization parameters remains a critical step in production [47].

Cross-linking techniques—chemical, physical, or enzymatic—are often applied to modulate degradation rate and mechanical performance. Non–cross-linked matrices generally integrate rapidly and favor early vascularization, while cross-linked variants offer extended structural stability and slower resorption. Table 3 compares cross-linking strategies and their influence on scaffold behavior. Chemical cross-linking prolongs membrane persistence but may limit cellular infiltration, whereas non–cross-linked matrices favor rapid vascularization and constructive tissue remodeling, which are key for periodontal soft-tissue healing.

**Figure 3:**
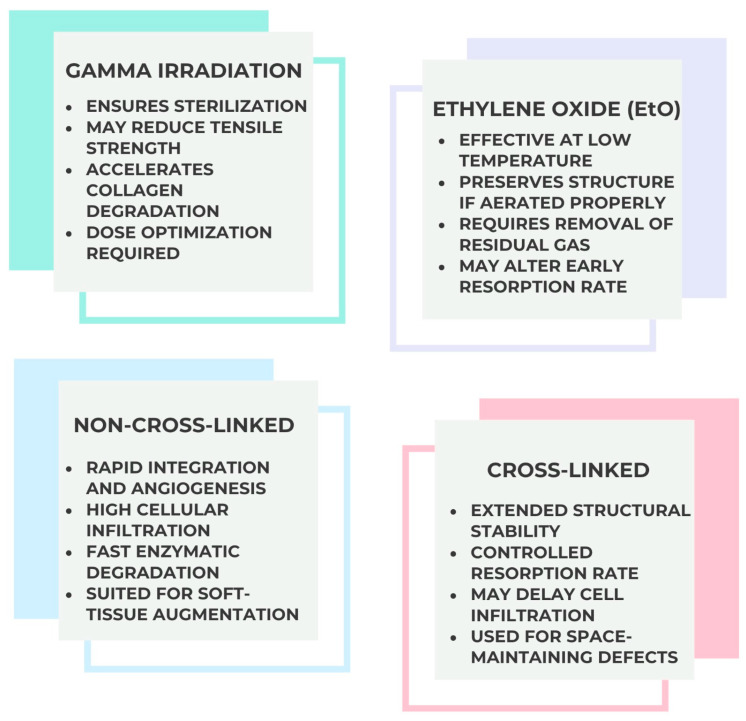
Sterilization for Porcine ECM Scaffold.

The selection of processing parameters is therefore tailored to the intended clinical application, balancing resorption dynamics with the regenerative requirements of the target defect. Figure 3 schematically summarizes sterilization pathways applied to porcine-derived scaffolds, emphasizing the trade-off between microbial inactivation and structural damage. In periodontal regeneration, maintaining collagen fibrillar architecture during sterilization is critical to prevent early membrane degradation and to support angiogenesis.

The three-dimensional organization of porcine ECM scaffolds represents a critical determinant of their biological performance. The intrinsic interconnectivity of collagen fibers and pores allows for optimal blood absorption, clot stabilization, and subsequent cellular infiltration [49]. The typical pore size, ranging between 50 and 200 µm, supports the migration of fibroblasts, endothelial cells, and osteogenic precursors, thereby facilitating early angiogenesis and tissue remodeling [50].

Upon hydration, the scaffold acquires viscoelastic properties that enable close adaptation to defect morphology without compromising dimensional stability. This balance between flexibility and mechanical integrity is particularly relevant in periodontal applications, where wound stability and space maintenance are prerequisites for predictable regeneration [51].

Functionally, the hydrated 3D network serves as a temporary extracellular niche, providing both mechanical support and biological signaling. The fibrillar structure acts as a reservoir for plasma proteins and cytokines, while collagen-bound peptides promote integrin-mediated cell adhesion [52]. Through gradual enzymatic degradation, the scaffold is progressively replaced by host-derived connective or mineralized tissue, in a process consistent with constructive remodeling [53].

The interplay between porosity, fiber orientation, and degradation kinetics dictates the scaffold’s capacity to sustain cell migration and matrix deposition. These architectural parameters, when properly balanced, transform the material from a passive filler into an active modulator of the regenerative cascade [54].

### 2.2. Classification and Main Types of 3D Porcine Biomaterials

The classification of porcine-derived three-dimensional (3D) biomaterials used in periodontal regeneration is primarily based on source tissue, processing technology, and intended clinical function. Each category exhibits distinct physicochemical and biological characteristics that determine its suitability for specific regenerative applications. According to source tissue, porcine ECM scaffolds originate mainly from dermis, small intestinal submucosa (SIS), or pericardium [55].

Dermal matrices exhibit dense type I collagen with low porosity, providing long-term stability for soft-tissue augmentation and gingival phenotype modification [56]. SIS scaffolds, in contrast, show a highly porous fibrillar architecture that promotes rapid cellular infiltration and angiogenesis, proving effective in intrabony, furcation, and peri-implant applications [57,58]. Pericardial collagen membranes are thin and multilayered, offering superior tensile strength and slow biodegradation, making them suitable for guided tissue regeneration [59,60].

Based on processing technology, matrices are classified as non–cross-linked or cross-linked. Non–cross-linked scaffolds preserve high biocompatibility and favor early vascularization but degrade faster, fitting for shallow or soft-tissue defects [61]. Cross-linked variants, treated chemically, enzymatically, or thermally, show prolonged resorption and improved dimensional stability for space-maintaining defects; however, excessive cross-linking may reduce cell penetration and bio resorption [62].

From a functional perspective, porcine 3D biomaterials are employed as barrier membranes for guided tissue/bone regeneration GTR/GBR procedures, soft-tissue matrices for mucogingival enhancement, composite scaffolds combining collagen with mineral or biologic additives and injectable or moldable forms designed for minimally invasive clinical use [63].

**Figure 4:**
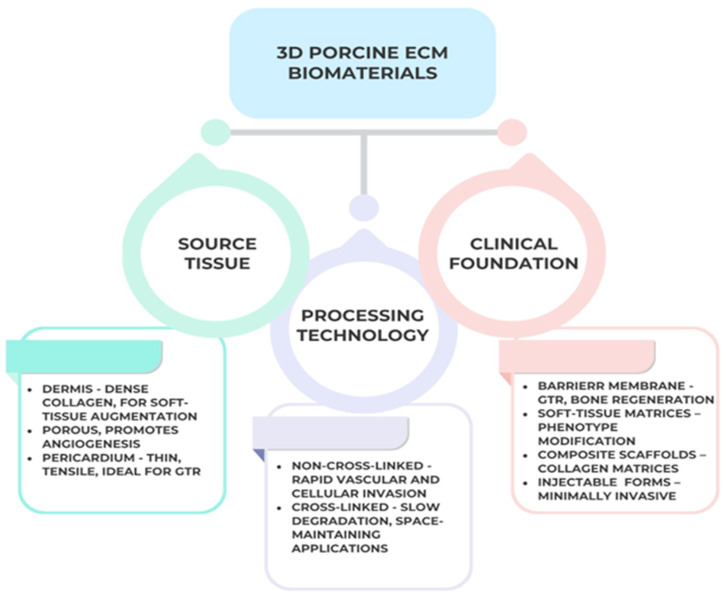
Classification of three-dimensional porcine ECM-derived biomaterials according to source tissue, processing technology, and clinical function. Created with BioRender.com.

This multifactorial classification highlights how structural origin and processing define the regenerative performance and clinical versatility of porcine ECM-based scaffolds. Figure 3 presents a functional classification of porcine ECM scaffolds based on tissue source and cross-linking type. This taxonomy highlights how dermal, SIS, and pericardial matrices exhibit distinct porosity and degradation kinetics, guiding clinicians toward indication-specific material selection in soft- and hard-tissue regeneration, as can be seen in Figure 4 below:

### 2.3. Evidence in Periodontology

The regenerative potential of porcine-derived extracellular matrix (ECM) scaffolds has been extensively evaluated in both preclinical and clinical settings, demonstrating their ability to promote the restoration of lost periodontal structures. Merging evidence has clarified several interrelated mechanisms through which porcine-derived ECM scaffolds support periodontal regeneration [64].

➢ Structural Stabilization and Controlled Degradation

Across animal and subcutaneous implantation models, porcine collagen membranes (mostly type I/III) show high biocompatibility and a wound response compatible with constructive remodeling [6]. In a mouse subcutaneous model, a non–cross-linked porcine collagen I/III membrane integrated predictably over 60 days with limited vascular ingrowth through the membrane, a mild mononuclear infiltrate, and preservation of barrier continuity-features aligned with GTR/GBR principles [65].

Many studies have been fundamental in elucidating the regenerative capacity of porcine-derived extracellular matrix scaffolds within the periodontal apparatus. Experimental models using animal subjects such as dogs, rats, and minipigs have provided insight into the ability of these materials to support osteogenesis, cementogenesis, and the re-establishment of functional periodontal ligament structures [66].

Two of the biggest problems with using collagen membranes for soft tissue augmentation in the oral cavity are volume instability and fast disintegration kinetics [67,68].

Collagenases found in periodontal bacteria, macrophages, and polymorphonuclear leukocytes undermine membrane stability [66,69]. To get the most out of them, efforts have been used to make collagen membranes stronger both mechanically and chemically. One such technique is exogenous collagen crosslinking, which is the process of making covalent links between collagen molecules using chemicals, physical processes, or biological mechanisms [70].

Chemical crosslinking procedures provide enhanced membrane stabilization; nevertheless, these techniques may provoke a detrimental host response, thereby undermining native tissue integration [71,72]. Physical and biological procedures avoid the risk of cytotoxicity, but they are far less effective than classic chemical methods [70]. Cross-linking makes the membrane stronger and more resistant to collagenase, which makes it last longer in vivo. This greater stability may give cells a steady signal to build rete ridges [73,74]. It is important to note that this resistance to degradation depends on the type of crosslinking used [73].

Collagen crosslinkers must have powerful crosslinking capabilities, excellent cytocompatibility, and rapid membrane degradation kinetics for these reasons. The regeneration capability of the membrane may be compromised due to cellular ingrowth, trans membranous vascularization, and a strong foreign body reaction brought on by the increased density that comes with substantial exogenous crosslinking [75,76]. Thus, whereas a highly crosslinked collagen membrane’s enhanced barrier function could work well for directed bone regeneration [77,78,79], it could be a disaster when it comes to enhancing soft tissues.

Prior studies have investigated the effects of non-crosslinked porcine-derived collagen matrix (Mucograft^®^) on soft tissue regeneration in patients with risk factors, such as smoking and intraoral manifestations of head and neck cancer treated with resection, which adversely affect their healing capacity post-periodontal surgery [80,81]. In these instances, Mucograft^®^ enhanced the width of keratinized tissue and the thickness of keratinized gingiva, facilitating total root coverage after 3 and 6 months [80,81]. In addition, the porcine-derived collagen membrane increased the diameter of the connected peri-implant gingiva and remained stable for six months [81].

A distinct clinical trial indicated that the oral epithelium displayed a keratinized stratum corneum with a developed keratin layer after 12 weeks, specifically concerning rete ridge shape [82].This study of Slavin et al. compared a novel, crosslinked porcine-derived collagen membrane (Zderm^TM^) to a non-crosslinked membrane (Mucograft^®^) and there were no significant variations in biocompatibility or gain of keratinised tissue length amongst the groups of membranes, except for the Zderm^TM^-associated qualitative improvements in rete ridge morphology [82].

➢ Soft-Tissue Regeneration and Phenotype Modulation Mechanism

Randomized clinical trials show that adding a porcine collagen matrix to a coronally advanced flap (CAF) can achieve clinically meaningful root coverage and soft-tissue thickening, offering an alternative to the connective tissue graft (CTG) while avoiding donor-site morbidity.

Cardaropoli et al. reported a prospective RCT where CAF+PCM performed as a viable substitute to CAF+CTG in Miller class I/II recessions, improving coverage and tissue phenotype [83]. Similarly, Moreira et al. compared CAF alone versus CAF+PCM for single recessions and found that the addition of a porcine matrix improved clinical outcomes versus flap alone [84].

Narrative syntheses concur that porcine acellular dermal matrix can increase keratinized tissue and thickness, while long-term equivalence with CTG still depends on defect characteristics and patient factors [85].

➢ Peri-Implant Soft-Tissue Augmentation

For peri-implant sites, xenogeneic volume-stable collagen matrices porcine-derived (VCMX) have been tested against CTG. A randomized controlled trial showed similar or greater soft-tissue volume gain at 90 days for VCMX compared with CTG; 5-year follow-up from the same cohort reported stable clinical and radiographic outcomes without clinically relevant differences in patient-reported measures [86].

Although there have been some promising preliminary findings from recent clinical case series using pre-hydrated porcine acellular dermal matrix, such as thickening of the buccal soft tissue at the time of implant insertion, these results should be confirmed in controlled trials to confirm their feasibility [87].

Though kinetics and tissue quality vary between materials and time, animal and ex vivo studies comparing porcine acellular dermal matrices with autologous CTG reveal early vascularization, keratinized layer development, and remodeling trajectories spanning 15–90 days. Differences in clinically observed early handling and integration can be better understood with the aid of these investigations [88].

Researchers have engineered resorbable barrier membranes out of porcine pericardium. These membranes have adjustable mechanics and degradation, with some variants being polyphenol-enhanced, which could lead to better space maintenance. These properties are important for periodontal intrabony/furcation regeneration, where the stability of the barrier is crucial. Current resorbable choices based on the pericardium have good biocompatibility, according to comprehensive assessments of barrier membranes in GBR/GTR. Although there is a strong foundation in materials science, clinical translation for periodontal abnormalities alone still necessitates indication-specific investigations.

Recent investigations have highlighted the enhanced regenerative potential of ECM scaffolds when used in combination with biologically active agents. The synergistic application of enamel matrix derivative (EMD), platelet-rich fibrin (PRF), and bone morphogenetic proteins (BMPs) with porcine-derived matrices have demonstrated superior outcomes in both soft and hard tissue regeneration compared to the use of the matrix alone.

➢ Porcine ECM combined with biologic agents (EMD, PRF, BMPs, or growth factors)

A recent study demonstrated that modifying a porcine-derived collagen matrix with a 24 h PRF exudate improved its bioactivity, suggesting enhanced adsorption and release of growth factors and improved cell behavior [89].

Lee et al. evaluated the adjunctive use of EMD with porcine-derived xenograft in one-wall intrabony defects, finding improved clinical outcomes over a 2-year follow-up [90]. Moreover, Pierfelice et al. reported an in vitro study where porcine collagen bone blended with collagen gel exhibited upregulation of BMP-2, ALP, and osteocalcin markers, indicating osteogenic potential of porcine scaffolds under biologic stimulation [91].

It seems that cell recruitment, differentiation, and growth factor release are all improved when porcine ECM scaffolds are combined with EMD, PRF, or osteoinductive factors, as opposed to when the scaffolds are used alone. Important but as-yet-unresolved issues include controlled delivery kinetics, scaffold functionalization, and dosage optimization.

➢ Immunomodulatory and Cellular Signaling Pathways

In addition to their structural function, ECM scaffolds generated from porcine have the ability to shape the host inflammatory response and direct tissue remodeling through their immunomodulatory actions. Rather than encouraging a fibrotic healing environment, new research shows that these matrices encourage a change in macrophage polarization from the pro-inflammatory M1 phenotype to the pro-remodeling M2 phenotype [92].

Cicuéndez et al. compared decellularized human and porcine adipose-derived matrices and demonstrated that scaffold source and processing strongly influence macrophage polarization and cytokine release profiles [93].

Similarly, Keane et al. emphasized that incomplete decellularization leading to residual cellular remnants can elicit a sustained M1-type inflammatory reaction and impair regenerative integration [94].

To further enhance mesenchymal cell adhesion and modulate immunological signaling toward tissue regeneration, the processing of porcine ECM scaffolds must preserve native bioactive components, including GAGs and integrin-binding motifs such as arginine–glycine–aspartate (RGD) sequences [95,96]. These molecular domains are crucial for mediating cell–matrix interactions, enabling fibroblasts, endothelial cells, and osteoblasts to attach, migrate, and differentiate appropriately within the regenerative microenvironment. The retention of these cues during scaffold manufacturing supports cellular recruitment, angiogenesis, and the deposition of host-derived extracellular matrix, thereby promoting biologically active healing [97,98].

Current research substantiates that porcine-derived extracellular matrix (ECM) scaffolds provide a physiologically compatible and functionally flexible category of biomaterials in periodontal and peri-implant regeneration. Preclinical and clinical investigations consistently demonstrate their capacity to enhance cell adhesion, angiogenesis, and guided tissue integration while lowering surgical morbidity compared with autogenous grafts [99].

Maintaining the structure of collagen and the binding sites for integrins during decellularization makes it easier for mesenchymal cells to move in and out and keeps the immune system in check, which leads to constructive remodeling instead of fibrotic encapsulation [100]. These characteristics result in clinically advantageous soft-tissue shape stability and improved defect closure, especially when utilized in conjunction with biologics like EMD or PRF.

Recent evidence underscores that the regenerative behavior of porcine extracellular matrix (ECM) scaffolds extends beyond their mechanical and structural roles, involving active immunomodulatory signaling that shapes the healing cascade [101]. Following implantation, ECM-derived degradation products and preserved integrin-binding motifs influence the host immune microenvironment by regulating macrophage polarization. A transient M1 inflammatory phase, responsible for initial debridement and microbial control, is progressively replaced by a dominant M2 phenotype associated with the secretion of anti-inflammatory cytokines (IL-10, TGF-β) and pro-regenerative mediators such as VEGF and PDGF [102,103,104]. This transition promotes angiogenesis, fibroblast migration, and matrix remodeling, leading to constructive rather than fibrotic healing.

Clinically, these immunomodulatory effects explain the favorable integration of porcine collagen matrices, which rarely elicit foreign-body reactions and support stable soft-tissue volume and keratinized gingiva over time [105]. The controlled degradation rate and balanced immune response allow for improved vascularization, epithelial sealing, and reduced postoperative inflammation, particularly when such matrices are combined with biologic adjuncts like enamel matrix derivatives or platelet-rich fibrin [106].

By linking molecular immune mechanisms with clinical outcomes, porcine ECM scaffolds emerge as bioactive therapeutic tools rather than passive barriers, contributing to predictable periodontal and peri-implant regeneration with minimal patient morbidity.

➢ Comparative Appraisal of Porcine ECM Scaffolds versus Xenogeneic, Allogenic, and Synthetic Alternatives

While porcine-derived extracellular matrix (ECM) scaffolds demonstrate remarkable biocompatibility and regenerative potential, their clinical value becomes more evident when critically contrasted with other xenogeneic, allogenic, and synthetic substitutes.

Compared with other xenogeneic matrices, such as bovine or pericardial collagen membranes, porcine scaffolds generally show faster vascularization and more dynamic cellular infiltration, favoring soft-tissue integration and remodeling [107]. However, bovine-based and pericardial variants often display greater tensile strength and slower resorption, which can be advantageous for space maintenance in guided bone regeneration but may limit early host–cell migration if heavily cross-linked [108]. Relative to allogenic acellular dermal matrices, porcine ECM offers comparable gains in keratinized tissue and gingival thickness, with the added benefit of more consistent availability and reduced regulatory and ethical constraints. Nevertheless, human-derived matrices sometimes provide superior pliability and handling in mucogingival applications [109].

When compared with synthetic barriers—such as expanded polytetrafluoroethylene (ePTFE) or resorbable polymeric membranes—porcine ECM scaffolds exhibit clearly higher intrinsic bioactivity due to preserved integrin-binding domains and glycosaminoglycans. Synthetic materials, while excellent for maintaining space and barrier function, lack biochemical signaling properties and may provoke a stronger foreign-body response or require removal if non-resorbable [110].

Overall, porcine-derived scaffolds strike a balance between biologic performance and clinical manageability: they support rapid angiogenesis, controlled degradation, and constructive remodeling with minimal morbidity. Their main limitations remain variability in degradation kinetics and mechanical strength compared with more rigid cross-linked or synthetic membranes [111].

The choice among these materials should therefore depend on defect morphology and therapeutic goals—favoring porcine ECM in soft-tissue phenotype modification and containing intrabony defects and reserving more stable xenogeneic or synthetic barriers for extensive or non-contained regenerative procedures.

A comparative summary of scaffold defects and used in periodontal and peri-implant regeneration is presented below. Table 4 highlights the biological, mechanical, and clinical performance features that distinguish porcine ECM scaffolds from allogenic, xenogeneic, and synthetic alternatives, summarizing their respective advantages, limitations, and preferred indications.

Beyond qualitative comparisons, integrating key physicochemical and biological parameters reported across studies can provide a quantitative overview of scaffold performance. Table 5 synthesizes representative values describing mechanical integrity, degradation dynamics, and biological responsiveness of porcine ECM scaffolds versus alternative materials.

Among the clinically validated matrices, Mucograft^®^, Zderm^®^, and VCMX^®^ represent the most extensively documented systems, each with distinct structural and biological profiles influencing clinical behavior. Mucograft is a non-cross-linked, bilayer porcine collagen matrix composed of Type I and III collagen with a compact barrier layer and a porous spongy layer. It supports rapid fibroblast migration and vascularization within 2–3 weeks, resulting in root coverage (CAL gain +2–3 mm) and keratinized tissue increase of 1.5–2.5 mm, comparable to connective tissue grafts (CTG) but with significantly lower patient morbidity. However, its faster degradation (8–10 weeks) may limit space maintenance in deep recessions or wide defects [112,113].

Zderm, an acellular dermal matrix (allogenic origin), provides favorable soft-tissue augmentation and color match due to its retained dermal collagen framework. Its integration is predictable and associated with good esthetic outcomes, yet variability in donor tissue and processing methods may influence consistency in remodeling and resorption rates [114].

VCMX (Volume-Stable Collagen Matrix) differs by its controlled cross-linking process, which stabilizes the fibrillar network and preserves three-dimensional volume for up to 20–24 weeks. It demonstrates high biocompatibility and stable peri-implant soft-tissue volume at long-term follow-up (≥5 years). The trade-off is slower vascular infiltration, requiring precise flap management to avoid early exposure [115,116].

Collectively, these findings indicate that non-cross-linked matrices like Mucograft favor rapid integration and reduced morbidity, whereas cross-linked systems such as VCMX or allogenic dermal substitutes (Zderm) provide enhanced volume stability in larger or high-tension sites. Understanding these material-specific biological dynamics enables clinicians to individualize regenerative strategies according to defect morphology and aesthetic demands.

A critical limitation across the available clinical evidence is the substantial heterogeneity in study design and case selection, which complicates cross-trial comparisons. Differences in defect morphology (single vs. multiple recessions; RT1–RT3; contained vs. non-contained intrabony defects; peri-implant tissue phenotype), surgical approach (CAF vs. tunnel; flap thickness; graft stabilization), and co-interventions (EMD, PRF, xenograft particulates) can independently influence root coverage, keratinized tissue gain, and volumetric stability. Material-related variability further contributes to dispersion: cross-linking status, thickness, and porosity modulate both degradation time and vascular ingrowth, altering early integration and space maintenance. Patient-level modifiers—gingival biotype, baseline KT width, smoking status, periodontal diagnosis and maintenance adherence—are inconsistently reported and rarely stratified, limiting external validity and the estimation of effect modifiers.

These sources of heterogeneity likely explain the long-term variability observed beyond 12–24 months, including soft-tissue shrinkage/relapse, differences in mucosal sealing around implants, and exposure/complication rates that are higher with stiffer or non-resorbable barriers.

Outcome measures are also heterogeneous: many trials prioritize short-term linear metrics (RC%, CAL, KT) without standardized volumetric endpoints (e.g., 3D intraoral scans) or patient-reported outcomes (pain, morbidity, aesthetics, satisfaction), which are central to graft-sparing indications.

Methodologically, future studies should report stratified results by defect class and biotype, prespecify core outcome sets (including a minimum clinically important difference), adopt blinded assessment and digital volumetrics, and ensure ≥36–60-month follow-up under documented supportive periodontal/peri-implant care. Such standardization would reduce interpretive uncertainty and allow more precise, indication-specific recommendations for scaffold selection.

## 3. Future Directions

While much has been learnt regarding the biological function of ECM scaffolds derived from pigs, there are still numerous crucial areas that want further investigation. To ensure uniform mechanical and biological outcomes across investigations, future research should focus on standardizing decellularization and sterilization procedures. Reducing immunogenicity while preserving the bioactive molecular domains crucial for constructive remodeling can be achieved by enhancing these processes.

Another major direction lies in the biofunctionalization and smart modification of ECM scaffolds. Combining porcine matrices with biologic agents such as enamel matrix derivatives (EMD), platelet-rich fibrin (PRF), bone morphogenetic proteins (BMPs), and controlled-release growth factors could enhance angiogenesis, osteogenesis, and host–scaffold integration. However, additional studies are needed to define optimal concentrations, delivery kinetics, and long-term stability of these hybrid constructs.

Future clinical and preclinical research should concentrate on molecularly characterizing cell–matrix interactions, particularly those related to macrophage polarization, stem cell recruitment, and critical signaling cascades. To obtain deeper insights into the complex immune regenerative environment associated with porcine ECM biomaterials, multi-omics methodologies, including transcriptomics, proteomics, and metabolomics, can be advantageous.

Long-term randomized controlled trials with standardized endpoints and radiographic, histologic, and patient-centered outcomes should be the main focus of clinical research to prove that these scaffolds are safe and predictable. In addition, designs that are particular to the tissue and the indication (for example, intrabony vs. peri-implant deficiencies) are necessary to customize the selection and use of biomaterials.

Recent high-level evidence further refines the indication spectrum of porcine-derived collagen matrices. In multiple recessions, SR/MAs confirm that while CTG remains the reference, collagen matrices can achieve clinically meaningful root coverage and soft-tissue thickening with reduced morbidity, supporting a patient-centered choice in suitable defects [117,118].

At implant sites, network and pairwise meta-analyses indicate that xenogeneic matrices provide measurable thickness gains, albeit typically lower than CTG, yet with favorable handling and patient acceptance; these data help tailor material selection to aesthetic demand, defect anatomy, and donor-site constraints [119,120].

Moreover, meta-analytic syntheses on PRF support its adjunctive use to enhance CAL/PD and bone fill, which is relevant when matrices are combined with biologics in intrabony and furcation defects [121,122].

Despite the overall favorable outcomes reported for porcine-derived biomaterials, a critical analysis of the available literature reveals several limitations, inconsistencies, and contradictory findings that must be acknowledged for a balanced interpretation. Methodological heterogeneity remains one of the main challenges: studies differ considerably in sample size, defect type, follow-up duration, and outcome measures, which complicate direct comparison and meta-analytical synthesis.

Moreover, variability in processing techniques—including decellularization protocols, sterilization methods, and the degree of residual cross-linking—can influence scaffold bioactivity and host response, sometimes resulting in delayed remodeling, partial resorption, or localized inflammation [71,72,73,74].

Certain histological studies have reported limited osteogenic potential or incomplete integration when porcine matrices were excessively processed or chemically stabilized, suggesting that the preservation of native ECM structure is critical for biological performance. In contrast, other investigations have found no statistically significant advantage of porcine scaffolds over conventional xenografts or collagen membranes in bone fill or attachment gain [75,76]. These discrepancies may arise from differences in material formulation, surgical handling, or defect morphology rather than intrinsic biomaterial inefficacy.

Furthermore, the absence of standardized manufacturing protocols and long-term multicenter trials restricts the generalizability of current findings. Potential conflicts of interest and the limited availability of independent comparative studies further underline the need for critical evaluation of commercial claims. Addressing these issues through rigorous, standardized, and transparently reported research will be essential to confirm the reproducibility, safety, and clinical predictability of porcine-derived biomaterials in periodontal regeneration.

Finally, combining 3D bioprinting with computer modelling [101] could lead to the creation of ECM-based constructions that are tailored to each patient and mimic the structure of natural periodontal tissues. These kinds of personalized scaffolds could change the field of regeneration periodontology by giving researchers precise control over the shape, breakdown, and biological performance of the scaffolds.

Translational progress in the field of ECM-based scaffolds will depend on aligning biomaterial design with measurable clinical endpoints. Future investigations should integrate digital volumetric analysis to quantify soft-tissue thickness gain and dimensional stability, alongside standardized measures of clinical attachment level (CAL) improvement and keratinized tissue increase. For implant-related applications, endpoints such as mucosal sealing integrity, soft-tissue height preservation, and absence of peri-implant inflammation provide clinically meaningful benchmarks.

Beyond conventional healing indices, forthcoming trials should systematically include patient-reported outcomes (pain, morbidity, aesthetic satisfaction) to capture the full therapeutic impact of graft-sparing materials. Establishing core outcome sets and minimum clinically important differences will enable comparisons across studies and support evidence-based material selection.

Finally, interdisciplinary research combining material science, digital imaging, and long-term clinical monitoring (≥3–5 years) will be essential to validate how specific scaffold modifications—such as cross-linking density, porosity, or incorporated bioactive cues—translate into predictable, patient-centered clinical performance.

## 4. Conclusions

Functional three-dimensional porcine-derived collagen matrix biomaterials have become essential components in the contemporary paradigm of physiologically directed periodontal and peri-implant regeneration. The body of experimental and clinical evidence demonstrates that these xenogeneic scaffolds facilitate tissue integration by preserving collagen architecture, glycosaminoglycans, and integrin-binding motifs essential for cell adhesion, migration, and differentiation. When their decellularization and sterilization techniques are just right, they let the immune system respond in a controlled way that includes macrophage M2 polarization, angiogenesis, and constructive remodeling instead of fibrotic encapsulation.

Reducing patient morbidity and maintaining consistent volumetric and histological stability, porcine ECM scaffolds offer a physiologically advanced and therapeutically feasible alternative to autogenous transplants. Both the immune system and its healing can be aided by these matrices because of their ability to interact with crucial molecular pathways. Disparities in processing methods, an absence of long-term data, and the necessity for molecular standardization continue to impede their extensive usage in clinical settings.

Future progress in functional biomaterial research should concentrate on improving scaffold bioactivity, augmenting structural repeatability, and incorporating bioactive compounds or stem cell-derived factors to further enhance periodontal tissue regeneration. In the end, porcine ECM scaffolds show the change from passive barrier membranes to dynamic, functional biostructures that can control healing at the cellular and molecular levels. This is a big step towards predictable, physiologically driven regenerative dentistry.

Current evidence confirms that porcine-derived ECM scaffolds represent biocompatible and clinically effective biomaterials for soft- and hard-tissue regeneration, offering predictable integration and reduced morbidity compared with autologous grafts. However, the available studies are heterogeneous in design, defect classification, and follow-up, which limits cross-comparison and meta-analytic interpretation. Differences in processing protocols (cross-linking, thickness, sterilization) and patient selection variables (biotype, periodontal status, maintenance) introduce additional variability that can affect healing dynamics and volumetric stability.

Long-term outcomes beyond 24–36 months remain scarce, and few trials include standardized volumetric or patient-reported endpoints. Likewise, the immunomodulatory mechanisms that underpin scaffold-driven regeneration—particularly macrophage polarization and cytokine balance—are supported mainly by preclinical data and require stronger clinical correlation. Future research should therefore focus on long-term prospective studies using digital volumetrics, core outcome sets, and stratified analyses by defect type and biotype, as well as comparative trials integrating biological, mechanical, and patient-centered parameters.

Recognizing these current uncertainties underscores that porcine ECM scaffolds should be viewed as complementary, indication-specific tools within the broader spectrum of regenerative strategies, rather than universal replacements for autologous grafts. Continued refinement of biomaterial design and trial methodology will be essential to establish definitive, evidence-based clinical guidelines.

## Figures and Tables

**Table 1:** Microorganism inactivation ability of different sterilization techniques.

Category	Technique	Inactivation Level	Mycobacteria	Vegetative Bacteria	Bacterial Spores	Non-enveloped Virus	Enveloped Virus	Prions	Fungal
Heat	Heat treatment	High	✓	✓	✓	✓	✓	✓	✓
Irradiation	Gamma	High	✓	✓	✓	✓	✓	✓	
	UV	Medium	✓	✓					
Plasma	Plasma sterilization	High	✓	✓	✓	✓	✓	✓	✓
Chemical sterilization	Ethylene oxide (EtO)	High	✓	✓	✓	✓	✓	✓	✓

✓ for applicable. Data summarized from Liu et al., 2025 [35]; Rabbani et al., 2021 [44]; Pineda-Molina et al., 2024 [41].

**Table 2:** Advantages and disadvantages of sterilization techniques.

Method	Technique	Advantages	Disadvantages
Heat	Heat treatment	Simple, fast, effective, high penetration ability, no toxic residues	High temperature affects the structural properties of biodegradable polymers
Irradiation	Gamma	High penetration ability, low temperature, effective, easy to control, no residue	Induces structural property changes; dose rate lower than electron beams; long processing time
	UV	Fast, low temperature, low cost, no toxic residues	Not effective; induces structural and biochemical property changes in biodegradable polymers under long exposure
Plasma	Plasma	Low temperature, improved cell interaction, increased surface wettability; fast	May cause changes in chemical and mechanical properties; leaves reactive species
Chemical treatment	Ethylene oxide (EtO)	Effective, low temperature	Induces structural property changes; leaves toxic residue; flammable, explosive, carcinogenic

Data adapted from Gilpin and Yang, 2017 [38]; Jiwangga et al., 2024 [39]; Pineda-Molina et al., 2024 [41].

**Table 3:** Crosslinking Methods and Their Effects on ECM Properties.

Crosslinking Type	Process/Example	Effect on the Membrane	Clinical Outcome
Chemical crosslinking	Covalent links between collagen molecules using chemicals	Enhanced membrane stabilization; may provoke a detrimental host response, thereby undermining native tissue integration	Increased mechanical strength and resistance to collagenase; slower degradation; may impair soft-tissue enhancement [47]
Physical crosslinking	Physical processes	Avoids the risk of cytotoxicity, but far less effective than classic chemical methods	Maintains biocompatibility but limited stabilization effect [43]
Biological crosslinking	Biological mechanisms	Avoids the risk of cytotoxicity; less effective than chemical methods	Supports biocompatibility and tissue remodeling [48]
Non–crosslinked	Native collagen membrane	Integrated predictably over 60 days with limited vascular ingrowth through the membrane, mild mononuclear infiltrate, and preservation of barrier continuity	Compatible with constructive remodeling and guided tissue/bone regeneration principles [42]

Data compiled from Zhao et al., 2024 [42]; Whitehead et al., 2022 [43]; Moffat, et al., 2022 [47]; Golebiowska et al., 2024 [48].

**Table 4:** Critical comparison of scaffold classes used in periodontal/peri-implant regeneration.

Criterion	Porcine ECM (3D Collagen Matrices)	Allogenic (Acellular Dermis)	Other Xenogeneic (Bovine/Pericardial)	Synthetic (PTFE/ePTFE; Resorbables)
Intrinsic bioactivity	High (integrin motifs, GAGs; pro-remodeling)	Moderate–high (processing-dependent)	Moderate (source & cross-linking dependent)	None (inert barrier)
Immunogenicity	Low when well-decellularized; good M2 skew	Low–moderate; donor/process dependent	Low–moderate; with heavy cross-linking	Low–moderate foreign-body response
Vascularization & integration	Rapid, especially non-cross-linked	Good; predictable soft-tissue integration	Variable; reduced if heavily cross-linked	Limited (no bioactive cues)
Barrier/space maintenance	Moderate; time-limited in non-cross-linked	Moderate; indication-dependent	Moderate–high (pericardial, cross-linked)	High (especially PTFE/ePTFE)
Soft-tissue phenotype (KT/thickness)	Strong gains; CTG-sparing option	Strong gains; good aesthetics	Moderate; more suited to barrier roles	Limited direct effects
Bone/intrabony defects	Better combined with biologics/grafts	Possible adjunct; less primary choice	Suited for GBR (stability)	Very good for GBR (containment)
Exposure risk/impact	Lower; exposure often manageable	Moderate	Moderate	Higher impact for non-resorbables
Handling	Hydrates, drapes well; patient-friendly	Familiar; pliable	Thin, tensile; technique-sensitive	Rigid/firm (PTFE); variable (resorbables)
Need for removal	No	No	No	Often yes (non-resorbables)
Best-fit scenarios	Recession coverage, peri-implant phenotype thickening, contained intrabony with biologics	Mucogingival augmentation where donor-site morbidity must be avoided	GBR/GTR where longer barrier time is key	Non-contained/space-demanding GBR; when strict barrier control is needed

**Table 5:** Comparative synthesis of physicochemical and biological parameters reported for representative scaffold classes used in periodontal and peri-implant regeneration.

Parameter	Porcine ECM Scaffolds	Bovine/Pericardial Xenografts	Allogenic Dermal Matrix	Synthetic (PTFE/PLGA)	References
Tensile strength (MPa)	1.2–3.0 MPa (non-cross-linked collagen)	3.5–5.5 MPa (cross-linked pericardial)	2–4 MPa	8–12 MPa	[81,82,83,86,97]
Porosity (%)	65–85%	45–60%	55–70%	20–80%	[25,55,107]
Pore size (µm)	50–200 µm (open interconnected pores)	30–120 µm	70–180 µm	10–300 µm (variable)	[49,50,97]
Degradation time	8–12 weeks (non-cross-linked)	20–24 weeks (cross-linked)	10–16 weeks	Up to 6 months (resorbable)	[42,83,86,97,111]
Vascularization onset	2–3 weeks	4–6 weeks	3–5 weeks	Minimal	[81,82,86,97]
GAG/collagen retention (%)	45–60%	35–50%	40–55%	—	[25,39,40]
M2/M1 macrophage ratio	2.0–2.4 (pro-remodeling)	1.5–1.8	1.8–2.0	<1.0 (pro-inflammatory)	[92,93,94]
Clinical outcomes (CAL gain, KT increase)	CAL +2–3 mm; KT +1.5–2.5 mm	CAL +2 mm	CAL +2–3 mm	Variable	[83,84,86,99,101]

“—” for not applicable.

## Data Availability

No new data were created or analyzed in this study. Data sharing is not applicable to this article.

## Abbreviations

The following abbreviations are used in this manuscript:

GTR	Guided Tissue Regeneration
ECM	Extracellular Matrix
CAL	Clinical Attachment Level
PD	Probing Depth
3D	Three-Dimensional
PGs	Proteoglycans
GAGs	Glycosaminoglycans
dECM	Decellularized Extracellular Matrix
DAMPs	Damage-Associated Molecular Patterns
SIS	Small Intestinal Submucosa
SDS	Sodium Dodecyl Sulphate
GBR	Guided Bone Regeneration
PCM	Porcine Collagen Matrix
CAF	Coronally Advanced Flap
CTG	Connective Tissue Graft
VCMX	Xenogeneic Volume-Stable Collagen Matrices
EMD	Enamel Matrix Derivative
PRF	Platelet-Rich Fibrin
BMPs	Bone Morphogenetic Proteins
RGD	Arginine–Glycine–Aspartate
ePTFE	Polytetrafluoroethylene
SR/MA	Systematic Reviews and Meta-Analyses
